# Dementia Development during Long-Term Follow-Up after Surgical Aortic Valve Replacement with a Biological Prosthesis in a Geriatric Population

**DOI:** 10.3390/jcdd11050136

**Published:** 2024-04-28

**Authors:** Ivo Deblier, Karl Dossche, Anthony Vanermen, Wilhelm Mistiaen

**Affiliations:** 1 Faculty of Medicine, University of Antwerp, 2610 Antwerp, Belgium; ivo.deblier@zna.be (I.D.); karl.dossche@zna.be (K.D.); anthony.vanermen@zna.be (A.V.); 2 Department Cardiovascular Surgery, ZNA Middelheim General Hospital, 2020 Antwerp, Belgium

**Keywords:** surgical aortic valve replacement, dementia, predictors

## Abstract

Surgical aortic valve replacement (SAVR) with a biological heart valve prosthesis (BHV) is often used as a treatment in elderly patients with symptomatic aortic valve disease. This age group is also at risk for the development of dementia in the years following SAVR. The research question is “what are the predictors for the development of dementia?”. In 1500 patients undergoing SAVR with or without an associated procedure, preoperative (demographic, cardiac and non-cardiac comorbid conditions), perioperative (associated procedures, cross-clamp and cardiopulmonary bypass time) and postoperative 30-day adverse events (bleeding, thromboembolism, heart failure, conduction defects, arrhythmias, delirium, renal and pulmonary complications) were investigated for their effect on the occurrence of dementia by univariate analyses. Significant factors were entered in a multivariate analysis. The sum of the individual follow-up of the patients was 10,182 patient-years, with a mean follow-up of 6.8 years. Data for the development of dementia could be obtained in 1233 of the 1406 patients who left the hospital alive. Dementia during long-term follow-up developed in 216/1233 (17.2%) of the patients at 70 ± 37 months. Development of dementia reduced the mean survival from 123 (119–128) to 109 (102–116) months (*p* < 0.001). Postoperative delirium was the dominant predictor (OR = 3.55 with a 95%CI of 2.41–4.93; *p* < 0.00), followed by age > 80 years (2.38; 1.78–3.18; *p* < 0.001); preoperative atrial fibrillation (1.47; 1.07–2.01; *p* = 0.018); cardiopulmonary bypass time > 120 min (1.34; 1.02–1.78; *p* = 0.039) and postoperative thromboembolism (1.94; 1.02–3.70; *p* = 0.044). Postoperative delirium, as a marker for poor condition, and an age of 80 or more were the dominant predictors.

## 1. Introduction

Before the advent of percutaneous aortic valve implantation (TAVI), surgical aortic valve replacement (SAVR) was the only treatment that was symptom-relieving, cost-reducing and life-prolonging in patients with symptomatic aortic stenosis [1]. A biological heart valve (BHV) prosthesis was developed specifically for elderly patients because it produced sufficient lifelong acetylsalicylic acid as an antithrombotic protocol in the absence of other risk factors for thromboembolic events [2], while structural valve degeneration was much less prevalent in this age group [3]. However, elderly patients, generally, are also much more at risk of developing dementia in the years following SAVR. This condition reduces the quality of life and increases physical health problems considerably. With increasing life expectancy, the number of older people with vascular and neurodegenerative dementia is rising. At least 12 risk factors for dementia have been identified in earlier studies. Some of these are socio-economic in nature, while others are of cardiovascular origin, but age remains a major determinant [4]. Dementia, with its undesirable effects, might be expected during the long-term follow-up after SAVR in a significant portion of these old patients. This was already observed after CABG (coronary artery bypass graft) two decades ago [5]. For SAVR in an elderly population, data are still lacking. The research question therefore is the following: what are the predictors in the current era for the development of dementia during long-term follow-up after SAVR in this geriatric population?

## 2. Materials and Methods

### 2.1. Study Design and Patient Population

This is a retrospective file study of 1500 patients who underwent SAVR with a biological heart valve at a general hospital. The files under scrutiny included the referral letters from the general practitioners and the cardiologists. These letters contained the detailed anamnesis of the current symptoms and the past medical history. In none of these letters and reports was dementia was observed among the comorbid conditions. This is in line with the view that dementia is generally considered a contraindication for cardiac surgery. The extensive preoperative work-up at our hospital confirmed the absence of preoperative dementia. It also served as an indication that the mental capacity of these patients was sufficient to make informed decisions about their major healthcare choices. However, Mini-Mental State Exams (MMSEs) were not routine in the preoperative workups. The patients included were from 2006, just before the introduction of TAVI 2008, until 2017. The choice for a BHV prosthesis was made mostly because of age considerations. The BHV prosthesis can degenerate, but this process is slower in elderly patients. This type of prosthesis has the advantage that the risk for thromboembolic events is much lower compared with that using mechanical valves. Hence the use of difficult-to-regulate vitamin K antagonists as anticoagulant medication can be avoided. The preoperative and operative characteristics and the early postoperative outcomes that were scrutinized for this aim are listed within the tables.

### 2.2. Clinical Preoperative Definitions

Significant coronary, peripheral and carotid artery disease were defined as a stenosis of at least 50% or by the need for an invasive procedure. Atrial fibrillation and conduction defects of any type were diagnosed by electrocardiogram (ECG) or Holter monitoring. Left ventricular ejection fraction, peak and mean transvalvular gradient across the diseased valve were measured by echocardiography. Diabetes was defined by a fasting plasma glucose of >125 mg% or by the need for antidiabetic treatment. Chronic kidney dysfunction (CKD) was defined by a plasma creatinine of >1.3mg%. Surgery was considered “urgent” if an SAVR was needed at the index hospital stay (during which the diagnosis of aortic valve disease was established), while “emergent” referred to the need for an SAVR within 24 h. Arterial hypertension was diagnosed by repeated arterial blood pressure measurements of >140/90 mmHg or by being under chronic antihypertensive treatment. Acute myocardial infarction was documented on ECG and biochemically. Endocarditis was diagnosed according to the modified Duke’s criteria. Sudden ischemic neurologic deficits were documented by clinical exam and confirmed on medical imaging. Chronic obstructive pulmonary dysfunction (COPD) was defined by a forced expiratory value at 1 s (FEV1) that is lower than 70% of the predicted value. Pulmonary artery hypertension was defined as a pulmonary artery pressure (PAP) of over 30 mm Hg. Hyperlipidemia was recorded on referral letters or documented by laboratory values. Congestive heart failure (CHF) was defined as a prior admission for pulmonary edema.

### 2.3. Definitions of Operative Parameters and of Postoperative Adverse Events

Cardiopulmonary bypass (CPB) and cross-clamp times were recorded. Mitral valve repair included quadrangular resections, the placement of rings, and the repair of chordae. Procedures on the ascending aorta included the narrowing, broadening or replacement of the ascending aorta. Other procedures were the maze procedure and a partial septal resection. The length of stay (LOS) in an intensive care unit (ICU) and the duration of mechanical ventilation were recorded in days and hours, respectively. The LOS in ICU of more than one day and a need for ventilation of more than 8 h were considered to be prolonged. Thromboembolic events were defined as sudden neurologic deficits or ischemia in other organs and limbs. Bleeding events were hematuria, hematomas of soft tissues, hemarthrosis and cerebral hemorrhage. Conduction defects were diagnosed as any new or the progress of pre-existing conduction defects on ECG, while atrial fibrillation was recorded on ECG, also as any new or recurring event. Congestive heart failure was defined as a need for inotropic medication, pulmonary or peripheral edema, or the need for mechanical circulatory support. Pulmonary complications were documented by clinical or radiological signs of atelectasis and pneumonia. Acute kidney injury was defined as an increase in plasma creatinine of at least 0.3 mg% and reaching the threshold of 1.3 mg%. The outcome of the current investigation was dementia (vascular, Alzheimer or mixed type) during long-term follow-up. This condition was diagnosed by a psychogeriatric evaluation, repetitive low scores on the MMSE scale in non-acute settings, or the need for acetyl cholinesterase inhibitors in the context of a psychiatric report. Computer tomography (CT) and magnetic resonance imaging (MRI) had a supportive value by detecting cortex atrophy and white-matter lesions.

### 2.4. Statistical Analysis

A Kaplan–Meier statistical analysis with a log-rank test was used to assess the effect of preoperative and operative factors as well as of early postoperative events on the outcome of “development of dementia” during long-term follow-up. This also included the event-free rates at 5 and 10 years. For continuous variables, a Student’s *t*-test was used. The Kaplan–Meier analysis was also used to assess the effect of dementia on survival. Factors with a significant effect for the development of dementia were entered into a multivariate Cox’s proportional hazard analysis. Significant predictors were identified bys their odds ratio and the 95% confidence interval. The SPSS version 29 software was used for this purpose. This study was approved by the ZNA ethical committee under protocol N° 2656.

## 3. Results

The sum of the individual follow-up of the patients was 10,182 patient-years, with a mean follow-up of 6.8 years. Of the 1500 patients, 1406 (93.7%) left the hospital alive, and for 1233 patients, a follow-up with respect to the presence or absence of dementia could be obtained. Compared with the first 1000 patients, operated at our hospital between 1987 and 2006 [6], the percentage of patients aged over 80 (32.2%) and those with diabetes mellitus (24.9%) showed a considerable increase. The percentage of patients with a prior CABG (8.5%), COPD (27.3%), CKD (19.5%), or a prior episode of CHF (26.3%) plateaued from 2000 on. Only the presence of carotid artery disease (18.9%) had decreased. During long-term follow-up, 216/1233 or 17.2% of the patients had developed dementia. The mean time to develop dementia after operation was 70 ± 37 months. The mean survival for patients who developed dementia was 109 (102–116) months. For patients who did not develop this condition, the mean survival was 123 (119–128) months, which was significantly longer, with *p* < 0.001. In Tables 1–3, the first column indicates the factor under scrutiny. The second column shows the patients with dementia (with the factor “absent”; in %), and the third column shows the patients with dementia (and the factor “present”; also in %). The fourth column shows the *p*-value derived by the log-rank test. The significant factors were entered into a Cox’s proportional hazard analysis, with the results shown in Table 4, showing five predictors. 

### 3.1. Effect of Preoperative Factors

Age was the dominant preoperative factor with an effect: the mean age of patients at operation who developed dementia during follow-up was 77.5 ± 5.7 years for patients who did not, this was 74.7 ± 7.2 years, (*p* = 0.009). Of the preoperative factors (Table 1), age over 80 had the strongest effect on dementia. Established cardiovascular conditions such as coronary artery disease, pulmonary artery hypertension, atrial fibrillation, episodes of prior CHF or lower ejection fraction, and a high functional NYHA class all had a significant effect. Increased plasma creatinine also showed an effect. The severity of aortic valve disease (mean and peak transvalvular gradient and aortic valve area) as well as prior procedures such as SAVR (*p* = 0.130), carotid artery endarterectomy (*p* = 0.249), CABG (*p* = 0.581), percutaneous coronary intervention (*p* = 0.607) or permanent pacemaker implant (*p* = 0.841) had no significant effect on the outcome.

### 3.2. Effect of Operative Factors

Of the operative factors (Table 2), concomitant CABG and CPB time were the factors with the strongest effect on long-term dementia. Both factors were interchangeable factors, since coronary artery disease is a prerequisite for a CABG, which further leads to a prolonged CPB time. Small valves also had an effect.

### 3.3. Effect of Postoperative Factors

Of the early postoperative factors (Table 3), postoperative delirium was by far the most dominant factor for dementia; this was followed by acute renal injury (AKI). Postoperative low cardiac output syndrome, thromboembolism and bleeding also had a significant effect. Patients who had developed dementia during long-term follow-up had a significantly higher need for resources, such as the need for blood products, renal replacement therapy or a prolonged stay at an ICU. The only long-term adverse events associated with dementia were thromboembolism (26.5% vs. 15.6%, *p* < 0.001) and bleeding events (23.1% vs. 16.3%, *p* = 0.004). Other adverse cardiac events (CHF, conduction defect, atrial fibrillation, endocarditis) showed no significant association with a later development of dementia.

### 3.4. Long-Term Survival

**Figure 1:**
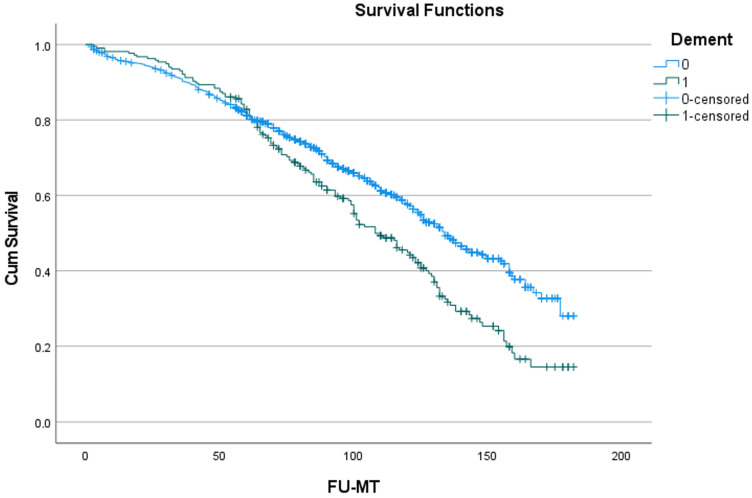
Effect of dementia on survival. Cum survival indicates the overall survival; Dement: dementia; blue curve “0”: survival without dementia; green curve “1”: survival with development of dementia. FU-MT: follow-up in months.

Figure 1 shows the effect of the development of dementia during follow-up on long-term survival. The development of dementia reduces survival significantly (*p* < 0.001). The curves diverge only after about 5 years. This trend can also be derived from the 5-year data (81.1 ± 1.2% vs. 80.9 ± 2.7%), which are very similar, and from the 10-year data (57.8 ± 1.9% vs. 44.9 ± 3.7%), which are more divergent.

### 3.5. Multivariate Analysis with Predictors for Dementia

Five independent predictors were identified by the Cox’s proportional hazard analysis (Table 4). Postoperative delirium had the highest odds ratio, and age over 80 was the second most important predictor. In descending order, the other predictors were preoperative atrial fibrillation, postoperative thromboembolism and concomitant CABG.

**Figure 2:**
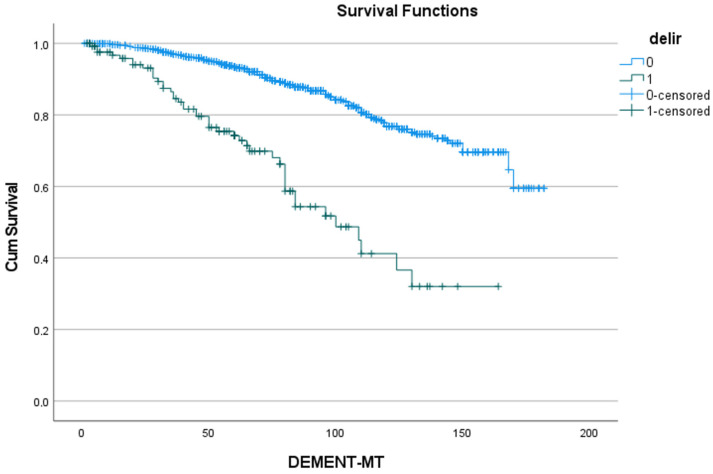
Effect of postoperative delirium on the development of dementia. Cum survival indicates the incidence of freedom from dementia; delir: delirium; blue curve “0”: freedom from dementia in patients without postoperative delirium; green curve “1”: freedom from dementia in patients with postoperative delirium; DEMENT-MT: follow-up in months.

**Figure 3:**
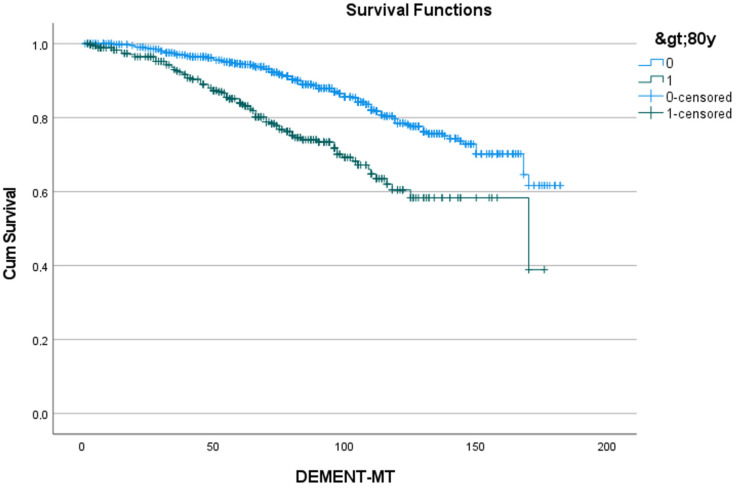
Effect of age > 80 years on the development of dementia. Cum survival indicates the incidence of freedom from dementia; blue curve “0”: freedom from dementia in patients younger than 80 years; green curve “1”: freedom from dementia in patients of 80 years and older; DEMENT-MT: follow-up in months.

**Figure 4:**
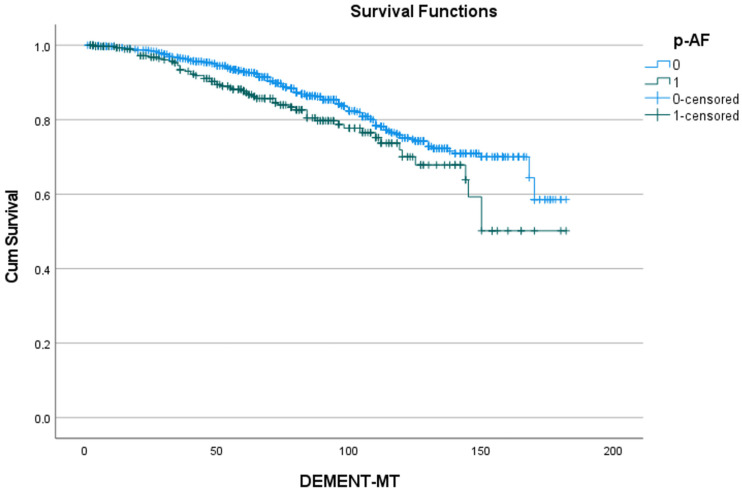
Effect of prior atrial fibrillation on the development of dementia. Cum survival indicates the incidence of freedom from dementia; p-AF: preoperative atrial fibrillation; blue curve “0”: freedom from dementia in patients without atrial fibrillation; green curve “1”: freedom from dementia in patients with atrial fibrillation; DEMENT-MT: follow-up in months.

**Figure 5:**
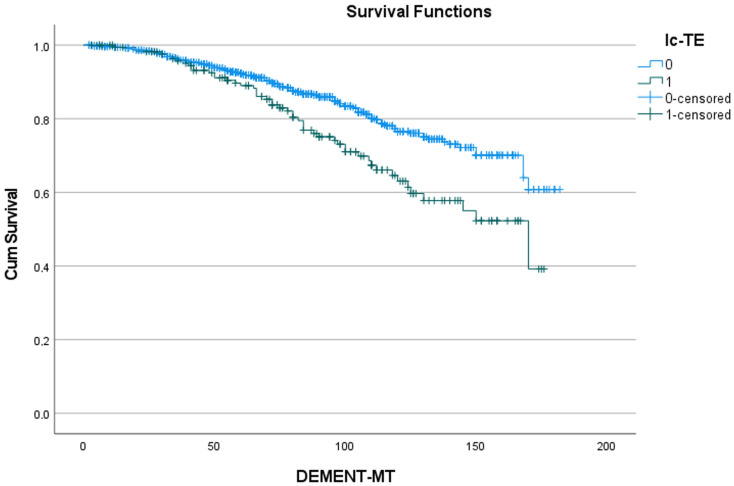
Effect of postoperative thromboembolism on the development of dementia. Cum survival indicates the incidence of freedom from dementia; lc-TE: postoperative thromboembolism; blue curve “0”: freedom from dementia in patients without postoperative thromboembolism; green curve “1”: freedom from dementia in patients with postoperative thromboembolism; DEMENT-MT: follow-up in months.

**Figure 6:**
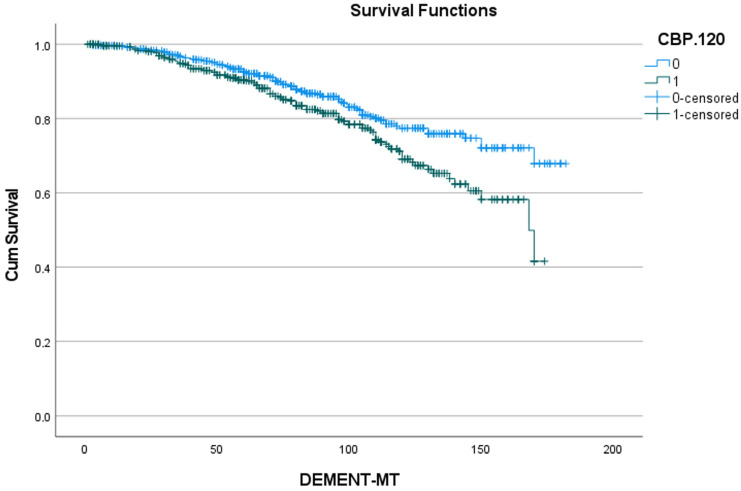
Effect of cardiopulmonary bypass time > 120 min on the development of dementia. Cum survival indicates the incidence of freedom from dementia; CPB.120 min: cardiopulmonary bypass time of more than 120 min; blue curve “0”: freedom from dementia in patients with a cardiopulmonary bypass time of less than 120 min; green curve “1”: freedom from dementia in patients with a cardiopulmonary bypass time of 120 min or more; DEMENT-MT: follow-up in months.

Table 5 shows the 5- and 10-year rates of freedom from dementia for the five predictors identified in Table 4, as well as the number of patients at risk. Figure 2, Figure 3, Figure 4, Figure 5 and Figure 6 show the associated survival curves with the effect of these predictors on dementia. The most divergent curves with the largest effect are those for delirium, followed by age above 80 and by postoperative thromboembolism. The shape of the curves also indicates that dementia rarely develops in the first year after SAVR.

## 4. Discussion

Dementia developed in 17.2% of the patients in the years following SAVR. The mean time to develop this condition was about 6–7 years. It is doubtful that surgery and anesthesia itself would have promoted or accelerated the development of dementia [7]. One large-scale meta-analysis found an association between general anesthesia and Alzheimer disease, but the results of the subgroup analyses were inconsistent, and publication bias was considered possible. The effect of surgery, and hence of the underlying condition, could be difficult to distinguish from the effect of anesthesia [8]. One prospective population-based cohort study could not associate the exposure to anesthesia in older adults to an increased risk for the development of dementia. High-risk surgery with general anesthesia, however, showed an increased risk for dementia. The surgery itself could act as a stressor, leading to delirium and subsequent dementia [9]. The need for high-risk surgery, however, could not be disentangled from the underlying condition needing this surgical correction. The current patient population was highly selected in this respect because of its type of heart disease (which compels the use of an extracorporeal circulation) and its high age. However, in one meta-analysis, which included over 90,000 patients, some form of preoperative cognitive impairment was detected in 19% of the patients who underwent a CABG [10]. In the current study, the previously identified [4] cluster of cardiovascular risk factors for dementia in the general population, such as smoking, diabetes, hypertension and obesity, were probably more common compared with the general population. Moreover, depression, deprived social contact, level of education, level of physical exercise, traumatic brain injury, air pollution, sleep disturbances and alcohol consumption as potential factors for dementia were not taken into account in the current study design. The current issues of interest were the identification of predictors for dementia during long-term follow-up after SAVR, which were postoperative delirium and high age, followed by three cardiovascular predictors.

### 4.1. The Effect of Dementia on Survival

Patients who developed dementia during long-term follow-up after SAVR in the current series had a mean age of 77.5 years, while the mean time to develop dementia after an operation was about 6–7 years. This indicated that dementia is usually diagnosed at a mean age of 82–84 years. This was in line with the results found in a large multicenter, population-based, prospective cohort study of individuals aged 65 years or older in community and residential settings. Survival after the diagnosis of dementia in this large series varied between 3.6 and 4.6 years, depending on gender, social engagement and level of education [11]. Our patients with dementia had a mean postoperative survival of 9 years, which indicates that the mean survival after the diagnosis of dementia was about 3 years, which was somewhat less compared with the aforementioned multicenter study. The current patient group were the survivors of a major and complex surgery for a potentially fatal heart disease, which has to be taken into account in any comparison with larger population groups. For patients without dementia, the mean postoperative survival was 10 years. The divergence in survival curves between those patients with and without dementia only became evident after 5 years of follow-up, which is about one year before dementia became apparent.

### 4.2. Delirium and the Use of Extracorporeal Circulation as Predictors for Dementia

Delirium was the strongest predictor for the development of dementia in the current series. Surgery and anesthesia are well-recognized stimuli in the development of postoperative delirium, especially after cardiac surgery. This event was observed in just over 10% of our patients, although this was without using a specific screening tool. With such a tool, this could increase to up to 24% [10]. In one prospective series, including patients with a mean age about fifteen years less than in the current population, cognitive decline was present in 53% of the patients after discharge after a CABG. There was an improvement at six months, but with a persistent decline, in 24% of the patients. This cognitive decline was found to have increased again at five years after the operation to a level of 42%. An early postoperative decline together with a higher age and a lower educational level predicted a long-term cognitive decline [12]. Such an initial improvement in cognitive function in the first months followed by a subsequent worsening was also found in a meta-analysis [10]. In another population with a mean age of 68 years, which was also considerable younger than the current patient group, a prevalence of dementia of 31% at 7.5 years after a CABG was observed, which was considerably higher compared with that in the general population. Univariate predictors were preoperative cognitive impairment and peripheral artery disease, but not postoperative cognitive dysfunction [12]. This differed from the prevalence of dementia in the meta-analysis of 7% five to seven years postoperatively, which was comparable to that in the general elderly population. However, the incidence of dementia was highly dependent on the length of the follow-up and could increase to 31% with a longer follow-up and the application of rigorous diagnostic testing. No analysis according to age or other risk factors was provided [10]. Delirium might be a harbinger for future dementia, but it also might be a marker for severe illness or organ dysfunction. Use of extracorporeal circulation is also a potentially strong factor in the development of postoperative delirium. For the current population, a long CPB time has been identified as a factor in the development of dementia, but only in a univariate analysis. Longer CPB times correlate with postoperative delirium and can occur in about 30% of patients [13,14]. This might be due to an increased release of chemokines, the complexity of surgery or an increased embolic load to the central nervous system [13]. The use of an ECC itself is not a factor for investigation in the current setting since SAVR cannot be performed without it. Moreover, the effect of extracorporeal circulation on cognitive function at 10 weeks and at 3 months remains a matter for debate [15,16]. In our current results, a prolonged CPB time, which is related to the need for a concomitant CABG, showed an increase in risk for dementia. In contrast, a population-based study in Sweden showed that in CABG patients who were compared with age- and gender-matched controls, there was no increase in the risk for dementia during postoperative follow-up. Only in younger patients, and especially in women, was a higher risk for delirium observed after 10 years [17]. The Swedish patient group is not necessarily comparable to the current one, since ours involved severe aortic valve disease, while the need for a concomitant CABG was a secondary issue. Moreover, in an orthopedic setting, dementia development within the first year after surgery has been described in patients suffering from postoperative delirium. This might, however, be due to a bias, where delirium may unmask a preoperative but undiagnosed cognitive decline. Delirium might lead to permanent brain damage and an acceleration of cognitive decline. Neurotoxicity and inflammatory changes could be shared mechanisms for delirium and dementia. Postoperative delirium and dementia seem to have a two-way relationship [18,19]. Postoperative delirium is, in a number of cases, a consequence of pre-existent mild cognitive impairment, which in itself could be a precursor to dementia. According to some, however, postoperative delirium is probably not related to a continuation of mild cognitive decline toward dementia [20]. In the current population, no mention was made in the referral letters of serious cognitive impairment before the SAVR. Preoperative mild cognitive impairment, however, was difficult to rule out, and could not, therefore, be included as a parameter in the current series.

### 4.3. The Effect of High Age

Age is a universally recognized factor for the development of dementia, which can also be observed in the current series. Age, cardiovascular disease and dementia have a somewhat complicated relationship. Dementia from all causes is more common with increasing age [20,21,22]. White-matter lesions on MRI due to atherosclerosis, myelin loss and gliosis play a role here [22]. After 60 years, the incidence of dementia doubles with each decade. Several cardiovascular factors promote the development of dementia. Treatment of these cardiovascular factors can improve life expectancy, but increasing the life-expectancy could make the development of dementia more likely. The population under investigation was a very select one, especially with respect to age: the first 400 patients in our institution who underwent SAVR with a biological valve between 1986 and 1998 had a median age of 73 years [23]. An increase in mean age and, particularly, of octogenarians was observed in the first 1000 patients who underwent SAVR in the period up to 2007, just prior to the introduction of TAVI [6]. This trend continued at least until 2016 and was observed even for patients 85 years and older [24]. About one-third of the patients in the current investigation consisted of octogenarians. The mean age of the patients at surgery was 75.7 ± 7.0 years. Diagnosis of dementia was made after a mean of 70 ± 37 months, indicating that dementia occurred at a likely mean age of about 82–84 years. This is in line with the estimation of dementia of all types in the general population of that age class that was made on 1 January 2015 in Flanders [25]: the prevalence of dementia for males and females aged between 80 and 84 years was 14.5% and 16.4%, respectively. The number of patients with dementia in the total Flemish population was 121.161 persons for both genders in 2015, and this was expected to rise to 134.818 in 2020. This expected increase of about 11.3% was faster than the relative growth of the general population, which was 2.8%. It is therefore safe to assume that these figures for the general population match very closely the prevalence of dementia observed in the current patient population. This is an indication that cardiac surgery, and more specifically SAVR, which always requires the use of an ECC, does not contribute to a significant degree to the development of dementia.

### 4.4. The Effect of Cardiovascular Factors

Atrial fibrillation (AF) increased the risk for thromboembolism (TE), which, in turn, could promote the development of dementia, but atrial fibrillation could also have an effect on dementia independently of TE. The duration of AF is of importance in this respect. There are different potential mechanisms: silent micro-embolic infarctions, detected only on MRI, cerebral hypoperfusion because of beat-to-beat variation in blood flow during AF [26], inflammation, diabetes and hypertension [27], as well as thromboembolism and stroke [28]. Dementia and cognitive dysfunction can occur in 25–30% of patients after ischemic stroke. However, the often-unknown pre-stroke cognitive status has to be taken into account, especially if other long-term risk factors exist. Hypoperfusion of the brain might also be one of the mechanisms in postoperative CHF. The latter has been identified only in a univariate analysis [21]. In the current patient series, preoperative AF and early postoperative TE were identified as independent predictors. TE during long-term follow-up was also associated with the development of dementia, but a relationship in time could not be established. Heart failure could not be identified as a predictor for dementia in the current series. Furthermore, insidious neurodegenerative processes could develop simultaneously [21]. Acute stroke and dementia might be linked through the vascular deposition of amyloid-beta, which impairs the balance between the deposition and clearance of this protein. The blood–brain barrier and perivascular space integrity breakdown, inflammation and hypoxia could increase the deposition of amyloid-beta, leading to synaptic dysfunction and, hence, cognitive decline [29]. This would be in agreement with white-matter degeneration, which is observed in vascular and other types of dementia [30].

### 4.5. The Effect of Chronic Kidney Dysfunction

Chronic kidney dysfunction (CKD) was the most important non-cardiac factor with an effect on the postoperative development of dementia in our series, but this factor was only apparent in a univariate analysis. In a recent meta-analysis, a significant relation was found for albuminuria, but this was less clear for the estimated glomerular filtration rate, serum creatinine and creatinine clearance. The mechanisms between CKD and dementia could include shared factors such as atrial fibrillation, stroke, diabetes, hypertension, age, smoking and hyperlipidemia. These factors are more prominent in both renal disease and dementia [31]. In one series, neurological damage and cognitive impairment were observed during acute renal injury (ARI) in critically ill elderly patients. Cardiac surgery patients were excluded in this setting [32]; nevertheless, ARI has been proven to be a serious problem after SAVR [33,34]. In a nationwide survey in Taiwan, an almost twice-as-high risk for long-term functional changes in the brain was observed in patients who had suffered a newly diagnosed episode of ARI. This event mediates oxidative stress and inflammation with capillary porosity. This damages the endothelium in the brain [33]. Nephrogenic factors such as oxidative stress, inflammation and vascular calcification are also held responsible for the development in patients with CKD [35]. Moreover, ARI and CKD are interrelated, whereby CKD can provoke ARI [6] after cardiac surgery. However, in the epidemiological study of Taiwan, development of dementia after ARI was found to be independent of CKD, but other unknown factors might have confounded the effect [34]. In the current series, ARI was the postoperative factor with the second greatest effect on the development of dementia. But this was only in a univariate analysis.

## 5. Limitations

There are several limitations to this study. No distinction has been made between the different types of dementia, nor between the different mechanisms in its development, which requires a thorough neurobiological investigation. This is a retrospective observational study, with all its inherent limitations. Preoperative screening for mild cognitive impairment by the use of MMSEs was not routine in the work-up. However, the inability to respond to a detailed anamnesis of past medical conditions, symptoms of the current disease and functional limitations could be indicative for further psychiatric evaluation. These inabilities were not found in the patient files, indicating that no patients with dementia were referred for SAVR by cardiologists, since dementia is considered a contraindication for cardiac surgery. The diagnosis of dementia during follow-up was dependent on the findings in the patient files. These findings included psychiatric reports, repeated MMSE evaluations in non-acute settings and the use of acetylcholinesterase inhibitors. However, dementia is a gradual disorder and can remain hidden for a long time because of its stigmatizing character. The event-free curves must be interpreted with caution. Furthermore, the sudden and unanticipated admission of patients into nursing homes might be responsible for a number of missed cases. Another limitation is the lack of information on the use of alcohol, social contact or lack thereof, and activities in daily life. Atrial fibrillation as a factor was grouped, and no distinction was made between paroxysmal, persistent and permanent AF. Delirium was defined as an acute and generally reversible disturbance of attention and cognition, with fluctuating symptoms such as confusion, restlessness, anxiety, irritability and sleep disturbances. However, some symptoms, such as social withdrawal, lack of attention and concentration, could easily have been missed if this was not actively investigated routinely. The incidence of delirium could therefore have been underestimated. Moreover, its definition could vary depending on the source.

## 6. Conclusions

Even with the need for extracorporeal circulation, there is little reason to believe that cardiac surgery and anesthesia itself lead to an accelerated development of dementia, since the prevalence of a postoperative development of dementia is close to the prevalence of dementia in the general population of the same age class. Postoperative delirium is the strongest predictor for the future development of dementia. However, delirium also seems to be a marker of existing comorbid conditions such as peripheral artery disease, cerebrovascular disease and, most of all, high age. This indicates that delirium itself is not the problem, but the underlying conditions are the culprit. These conditions include cardiovascular comorbidity, such as atrial fibrillation and thromboembolism. These are associated with aortic valve disease and SAVR. Our results confirm this view, since these factors were also identified as predictors for dementia. Renal disease has not been identified as an independent predictor for the development of dementia, but it remains highly significant in a univariate analysis.

## Figures and Tables

**Table 1:** Preoperative categorical factors with an effect on long-term dementia.

Factor	Factor Absent (%)	Factor Present (%)	*p*
Age > 80 years	128/879 (14.6)	83/354 (23.4)	<0.001
Coronary artery disease	68/491 (13.8)	143/742 (19.3)	0.001
Hyperlipidemia	118/777 (15.2)	90/437 (20.6)	0.002
Pulmonary artery hypertension	152/883 (17.2)	55/288 (19.1)	0.002
Atrial fibrillation	157/927 (16.9)	54/306 (17.6)	0.006
Congestive heart failure	159/949 (16.8)	52/283 (18.4)	0.006
Creatinine > 1.30 mg%	172/1019 (16.9)	38/212 (17.9)	0.008
NYHA functional class IV	116/692 (16.8)	25/134 (18.7)	0.001
Left ventricular ejection fraction	100/635 (15.7)	34/161 (21.1)	0.029
Cerebrovascular accident	185/1093 (16.9)	26/140 (18.6)	0.073
Urgent SAVR	173/1033 (16.7)	38/200 (19.0)	0.075
Diabetes mellitus	160/959 (16.7)	51/274 (18.6)	0.092
Peripheral artery disease	152/933 (16.3)	59/299 (19.7)	0.163
Emergent SAVR	196/1159 (16.9)	15/73 (20.5)	0.206
Endocarditis	210/1197 (17.5)	1/36 (2.8)	0.229
Acute myocardial infarction	179/1053 (17.0)	30/177 (16.9)	0.262
Male gender	88/477 (18.4)	123/756 (16.3)	0.308
Carotid artery disease	176/1020 (17.3)	35/213 (16.4)	0.353
Malignancy	183/1025 (17.9)	28/204 (13.7)	0.405
Conduction defects (all types)	147/830 (17.7)	64/403 (15.9)	0.547
Arterial hypertension	48/311 (15.4)	162/920 (17.6)	0.583
BMI > 30 kg/m^2^	156/872 (17.9)	52/343 (15.2)	0.600
FEV1 < 70% predicted	162/910 (17.8)	45/299 (15.1)	0.611
Smoking	169/973 (17.4)	40/243 (16.5)	0.636
Left ventricular hypertrophy	21/129 (16.3)	173/1030 (16.8)	0.878

BMI: body mass index; FEV1: forced expiratory volume at 1 s; NYHA: New York Heart Association.

**Table 2:** Operative factors with an effect on long-term dementia.

Factor	Factor Absent (%)	Factor Present (%)	*p*
Concomitant CABG	72/526 (13.7)	139/707 (19.7)	0.001
CPB time > 120 min	96/626 (15.4)	98/474 (20.7)	0.008
Smallest valve size	204/1212 (16.8)	6/19 (31.6)	0.041
Mitral valve repair	197/1165 (16.9)	14/68 (20.6)	0.088
Incomplete revascularization	190/1119 (17.0)	21/107 (19.6)	0.154
Ascending aorta procedure	197/1123 (17.5)	14/109 (12.8)	0.321
Concomitant CEA	209/1210 (17.3)	2/23 (8.7)	0.396
Cross-clamp time > 60 min	75/386 (19.4)	104/609 (17.1)	0.994

CABG: coronary artery bypass graft; CEA: carotid endarterectomy; CPB: cardiopulmonary bypass.

**Table 3:** Early postoperative adverse events with an effect on long-term dementia.

Factor	Factor Absent (%)	Factor Present (%)	*p*
Adverse events			
Delirium	169/1106 (15.3)	42/126 (33.3)	<0.001
Acute renal injury	155/956 (16.2)	56/274 (20.4)	<0.001
Low cardiac output syndrome	199/1163 (17.1)	12/69 (17.4)	0.002
Thromboembolism	201/1193 (16.8)	10/39 (25.6)	0.003
Bleeding	194/1157 (16.7)	17/75 (22.7)	0.006
Atrial fibrillation	129/756 (17.1)	82/476 (17.2)	0.243
Ventricular arrhythmias	205/1195 (17.2)	6/37 (16.2)	0.316
Pulmonary complication	187/1057 (17.7)	24/175 (13.7)	0.376
Myocardial infarction	210/1224 (17.2)	1/8 (12.5)	0.766
Conduction defect	173/1001 (17.3)	38/231 (16.5)	0.833
Endocarditis	211/1231 (17.1)	0/1 (0.0)	0.934
Need for resources			
>4 units of packed cells	158/1013 (15.6)	48/201 (23.9)	0.005
Reintervention	200/1193 (16.7)	11/40 (27.5)	0.012
Permanent PM implant	203/1197 (17.0)	8/36 (22.2)	0.021
Plasma derivatives	145/901 (16.1)	61/312 (19.6)	0.024
Thrombocyte concentrate	184/1086 (16.9)	22/127 (17.3)	0.024
Renal replacement therapy	203/1192 (17.0)	8/37 (21.6)	0.026
Length of stay in an ICU	152/896 (17.0)	58/335 (17.3)	0.045
Mechanical ventilation > 8 h	118/789 (15.0)	86/421 (20.4)	0.070

ICU: intensive care unit; PM: pacemaker.

**Table 4:** Identification of predictors for dementia by the Cox proportional hazard analysis.

Predictor	Odds Ratio	95%CI	*p*
Postoperative delirium	3.55	2.41–4.93	<0.001
Age > 80 years	2.38	1.78–3.18	<0.001
Preoperative atrial fibrillation	1.47	1.07–2.01	0.018
CP bypass time > 120 min	1.34	1.02–1.78	0.039
Postoperative thromboembolism	1.94	1.02–3.70	0.044

95%CI: 95% confidence interval; CP: cardiopulmonary.

**Table 5:** Kaplan–Meier analysis of the 5 and 10 y event-free rates for dementia.

Factor		Factor Absent (n)	Factor Present (n)	*p*
Delirium	5 y	93.4 ± 0.88 (839)	74.2 ± 4.3% (61)	<0.001
	10 y	77.4 ± 2.3% (132)	36.6 ± 9.7% (8)	
Age > 80 years	5 y	94.5 ± 0.8% (685)	84.0 ± 2.1% (216)	<0.001
	10 y	78.5 ± 2.0% (209)	60.5 ± 4.2% (36)	
Postoperative thromboembolism	5 y	92.3 ± 0.9% (760)	89.0 ± 2.5% (124)	<0.001
	10 y	76.5 ± 1.9% (202)	63.1 ± 4.8% (35)	
CP bypass time > 120 min.	5 y	92.5 ± 1.1% (483)	90.2 ± 1.4% (333)	0.005
	10 y	79.9 ± 2.5% (112)	69.1 ± 3.0% (102)	
Prior atrial fibrillation	5 y	92.8 ± 0.9% (709)	87.7 ± 2.9% (192)	0.016
	10 y	75.1 ± 2.0% (206)	70.0 ± 4.3% (31)	

CPB: cardiopulmonary; n: number of patients at risk.

## Data Availability

These results have been derived from a multipurpose database, from which several more publications will be derived. These data are not yet publicly available.
